# Heightened levels of plasma growth differentiation factor 15 in men living with HIV

**DOI:** 10.14814/phy2.15293

**Published:** 2022-05-04

**Authors:** Neeti Agarwal, Claudia E. Ramirez Bustamante, Huaizhu Wu, Reina Armamento‐Villareal, Jordan E. Lake, Ashok Balasubramanyam, Sean M. Hartig

**Affiliations:** ^1^ 3989 Division of Diabetes, Endocrinology, and Metabolism Baylor College of Medicine Houston Texas USA; ^2^ 3989 Atherosclerosis and Lipoprotein Research Baylor College of Medicine Houston Texas USA; ^3^ Center for Translational Research on Inflammatory Diseases Michael E DeBakey VA Medical Center Houston Texas USA; ^4^ Division of Infectious Diseases Department of Internal Medicine McGovern Medical School University of Texas Health Science Center Houston Texas USA; ^5^ 3989 Department of Molecular and Cellular Biology Baylor College of Medicine Houston Texas USA

**Keywords:** ART, energy balance, GDF15, HIV, metabolic syndrome

## Abstract

Plasma biomarkers that reflect energy balance disorders in people living with HIV (PLWH) remain limited. Growth differentiation factor 15 (GDF15) abundance in plasma of mice and humans induces negative energy balance but also becomes highly elevated in obesity and other metabolic diseases. We sought to compare plasma GDF15 levels in PLWH and HIV‐negative persons and mouse models expressing the HIV accessory protein Vpr (that recapitulate HIV‐associated metabolic disorders) and determine their relationship to metabolic parameters. We measured liver *Gdf15* mRNA levels and plasma GDF15 levels in male Vpr mice and littermate controls. In parallel, we analyzed plasma GDF15 levels in 18 male PLWH on stable, long‐term antiretroviral therapy and 13 HIV‐negative men (6 healthy controls and 7 with metabolic syndrome). Plasma GDF15 levels were correlated with anthropometric and immune cell parameters in humans. Gene expression analysis of Vpr mouse liver demonstrated elevated *Gdf15* mRNA. Plasma GDF15 levels were also higher in Vpr mouse models. Levels of plasma GDF15 in PLWH were greater than in both HIV‐negative groups and correlated positively with the CD4/CD8 T cell ratio in PLWH. Plasma GDF15 levels correlated positively with age in the HIV‐negative subjects but not in PLWH. Since GDF15 levels predict fatty liver disease and energy balance disorders, further studies are warranted to determine the effect of GDF15 in mediating the metabolic disturbances that occur in Vpr mice and PLWH.

## Graphical abstract

Growth differentiation factor 15 (GDF15) abundance in plasma of mice and humans induces negative energy balance but also becomes highly elevated in obesity and other metabolic diseases. We discovered that GDF15 levels are elevated in the plasma of persons living with HIV (PLWH). Since GDF15 levels predict fatty liver disease and energy balance disorders, further studies are warranted to determine the effect of GDF15 in mediating the metabolic disturbances that occur in mouse models and PLWH.

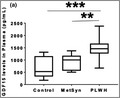

## 1 INTRODUCTION

Persons living with HIV (PLWH) accumulate systemic metabolic alterations (Carr et al., 2000), the cardinal features of which include intra‐abdominal fat accumulation, fatty liver disease, dyslipidemia, insulin resistance, and diabetes. The high prevalence of metabolic syndrome (MetSyn) in PLWH may relate to adipose tissue abnormalities and accelerated abdominal fat accumulation described among this group (Grant et al., 2016). Notably, compared to the general population, fatty liver disease in PLWH exhibits a more aggressive clinical course with a high frequency of non‐alcoholic steatohepatitis (NASH) and fibrosis (Fourman et al., 2021; Vodkin et al., 2015). Treatment of these manifold defects using conventional lipid‐lowering or insulin‐sensitizing drugs and lifestyle interventions has been minimally effective (Carr, 2003). Despite the critical need, biomarkers of MetSyn in PLWH remain lacking and no therapies for HIV‐associated fatty liver disease exist (Alkhouri et al., 2021).

Growth differentiation factor 15 (GDF15) is a circulating peptide that exerts weight‐lowering effects when administered to rodents and non‐human primates (Emmerson et al., 2017; Mullican et al., 2017). Intracellular stress drives the expression of GDF15 in the kidney, liver, skeletal muscle, and some immune cells. Other factors that elevate GDF15 include mitochondrial dysfunction (Coll et al., 2020; Hsiao et al., 2000; Keipert & Ost, 2021; Paralkar et al., 1998; Wang et al., 2021), the integrated stress response (Coll et al., 2020; Patel et al., 2019), and AMP‐associated protein kinase (AMPK) activation (O'Neill et al., 2011). Consequently, GDF15 expression is upregulated in chronic inflammatory diseases (Moon et al., 2020), cardiovascular disease (Adela & Banerjee, 2015; Wang et al., 2021), non‐alcoholic fatty liver disease (NAFLD) (Li et al., 2018) and diabetes (Adela & Banerjee, 2015; Hung et al., 2021; Wang et al., 2021).

Viral factors are central to the pathophysiology of metabolic dysfunction in PLWH (Dhurandhar, 2011; Lyons et al., 1982; Ramesh & Sanyal, 2004). We previously showed that the HIV accessory protein viral protein R (Vpr) circulates in the blood of PLWH even in the presence of viral‐suppressive antiretroviral therapy (ART). We also demonstrated that forced expression of Vpr in mice recapitulates essential features of metabolic disorders in PLWH, including hyperlipidemia, dysregulated adipose tissue function, and NAFLD (Agarwal et al., 2013; Agarwal et al., 2017; Agarwal et al., 2021). Mitochondrial dysfunction caused by HIV‐related factors and ART likely contribute to metabolic deterioration in PLWH (Sun et al., 2019), but the endocrine arms of these responses remain unclear. Along these lines, we explored GDF15 levels in the liver and serum of male Vpr mice and littermate controls. We also hypothesized GDF15 would be increased in the plasma of stable ART‐treated PLWH relative to HIV‐negative controls with and without MetSyn. Hence, we measured GDF15 levels in Vpr mice and PLWH and determined their relationships with immune reconstitution and available metabolic measures.

## 2 MATERIALS AND METHODS

### 2.1 Study design

We report data from male subjects who participated in studies of metabolic function and comorbidities as described below. In addition, we report data from 14 to 16 week‐old male mice. Human protocols were approved by the Institutional Review Board of Baylor College of Medicine, the University of California, Los Angeles, and the University of Texas Health Science Center, Houston. Animal protocols were approved by the Baylor College of Medicine Institutional Review Board and Institutional Animal Care and Use Committee.

### 2.2 Vpr transgenic (Vpr‐Tg) mice

FVBN mice expressing phosphoenolpyruvate carboxykinase (*Pepck*) promoter‐driven Vpr in the liver and adipose tissue under the control of a tetracycline‐repressible (tTA) system were constructed at the NIH (Balasubramanyam et al., 2007).

### 2.3 Synthetic Vpr

Synthetic Vpr (sVpr) was produced by solid‐state peptide synthesis, purified, characterized by sequencing and mass spectrometry, and compared to viral Vpr by SDS‐PAGE and immunoblotting (Henklein et al., 2000). Continuous delivery of sVpr or vehicle (sterile water) was achieved using Alzet pumps (Durect, Cupertino, CA). These were implanted subcutaneously in wild‐type FVBN mice with a delivery rate of 0.25 μl/h to administer 5 μg of sVpr/24 h for 14 days (Agarwal et al., 2013; Agarwal et al., 2017; Agarwal et al., 2021; Balasubramanyam et al., 2007).

### 2.4 Human plasma samples

De‐identified samples from a completed, IRB‐approved study of men with HIV at the University of California, Los Angeles, and the University of Texas Health Science Center Houston were used as convenience samples. All participants provided written informed consent to use residual samples for future comorbidities research studies. General inclusion criteria included HIV‐positive serostatus, male sex, age ≥ 50 years old, and HIV‐1 RNA < 200 copies/ml on stable ART for ≥24 weeks (Netanya et al., 2018). These PLWH were non‐diabetic and normoglycemic by fasting glucose measurements. Many were taking lipid‐lowering therapy. De‐identified samples of the HIV‐negative healthy control subjects and HIV‐negative subjects with MetSyn were obtained from Baylor College of Medicine IRB‐approved studies. The healthy control subjects had no chronic illnesses, were not taking any medications, were normoglycemic, and met ≤2 of the criteria for MetSyn. The subjects with MetSyn were also normoglycemic and not taking any medications but met ≥3 non‐glycemic criteria for MetSyn: waist circumference ≥ 102 cm, triglyceride levels ≥ 150 mg/dl, HDL‐C < 40 mg/dl, and SBP ≥ 130 mm Hg or DBP ≥ 85 mm Hg. All plasma samples were stored at −80°C until biomarker measurement. All PLWH were male, 67% White, 11% African, American, 11% Hispanic, and 11% Asian, aged 59 ± 1.73 years (mean ± SE), with a BMI of 27.1 ± 1.32 kg/m^2^ (Table 1). All healthy control subjects were male, 67% White, 33% Hispanic, with a BMI of 24.55 ± 1.23 kg/m^2^, aged 49 ± 7 years. All MetSyn subjects were male, 86% White, 14% African American, aged 61 ± 1.61 years (mean ± SE), with a BMI of 30.65 ± 0.87 kg/m^2^.

**TABLE 1:** Characteristics of participants at baseline

Characteristic	PLWH	HIV‐negative healthy controls	HIV‐negative metabolic syndrome patients	*p*‐value
Number of subjects	18	6	7	
Age (years)	59 ± 1.73	49 ± 7	61 ± 1.61	HC vs. PLWH =0.27 MetSyn vs. PLWH =0.61
Male sex (number)	18	6	7	
Ethnicity				
White	12	4	5	
African American	2	0	2	
Asian	2	0	0	
Hispanic	2	2	0	
BMI (kg/m^2^)	27.11 ± 1.32	24.55 ± 1.23	30.65 ± 0.87	HC vs. PLWH =0.81 MetSyn vs. PLWH =0.08

Note

All PLWH were normoglycemic on chronic antiretroviral therapy. All HIV‐negative subjects were normoglycemic, on no medications. Non‐glycemic MetSyn criteria included: Waist circumference ≥102 cm, triglyceride levels ≥150 mg/dl, HDL‐C < 40 mg/dl, SBP ≥130 mm Hg, or DBP ≥85 mm Hg. MetSyn was defined by the presence of ≥3 of these criteria. HC = healthy control. Values represent mean ± SD.

### 2.5 mRNA levels

Total RNA was extracted from livers of mice using the Trizol method (Invitrogen, Waltham, MA), transcribed using the RNA‐to‐cDNA kit (Applied Biosystems, Grand Island, NY), and PCR performed using the TaqMan^®^ probe‐based assay and Universal PCR Master Mix with an ABI 7000 Real‐Time PCR System (Applied Biosystems, Grand Island, NY). mRNA expression levels of *Gdf15* were measured and normalized to *Pgk1* mRNA expression.

### 2.6 GDF15 plasma analysis

GDF15 concentrations in plasma samples were measured by ELISA (Abcam, Milpitas, CA) following the manufacturer's protocols.

### 2.7 Statistics

The primary endpoints were levels of GDF15 in men living with HIV compared to males with and without MetSyn, and in male Vpr‐Tg and sVpr compared to wild‐type littermates. Secondary endpoints included liver GDF15 mRNA expression and its correlation with clinical parameters such as age, CD4, and CD8 counts. We used convenience samples from male healthy, MetSyn, and HIV subjects available to us. Thus, we did not calculate a sample size for the human protocol. Sample sizes for animal studies were based on our previous data regarding the effects of Vpr on adipose tissue and the liver (Agarwal et al., 2013; Agarwal et al., 2017; Agarwal et al., 2021). Descriptive statistics of demographic characteristics are presented as median with interquartile range (IQR) and as mean and standard deviation when appropriate. Comparisons between groups were made using Mann‐Whitney *U*. Correlation analyses were performed using Spearman's test. Linear regression analysis was performed relating GDF15 plasma levels to various demographic, anthropometric, and biochemical parameters in PLWH, control, and MetSyn groups. *p* < 0.05 was considered significant.

## 3 RESULTS

### 3.1 Vpr mice express elevated levels of Gdf15

**FIGURE 1:**
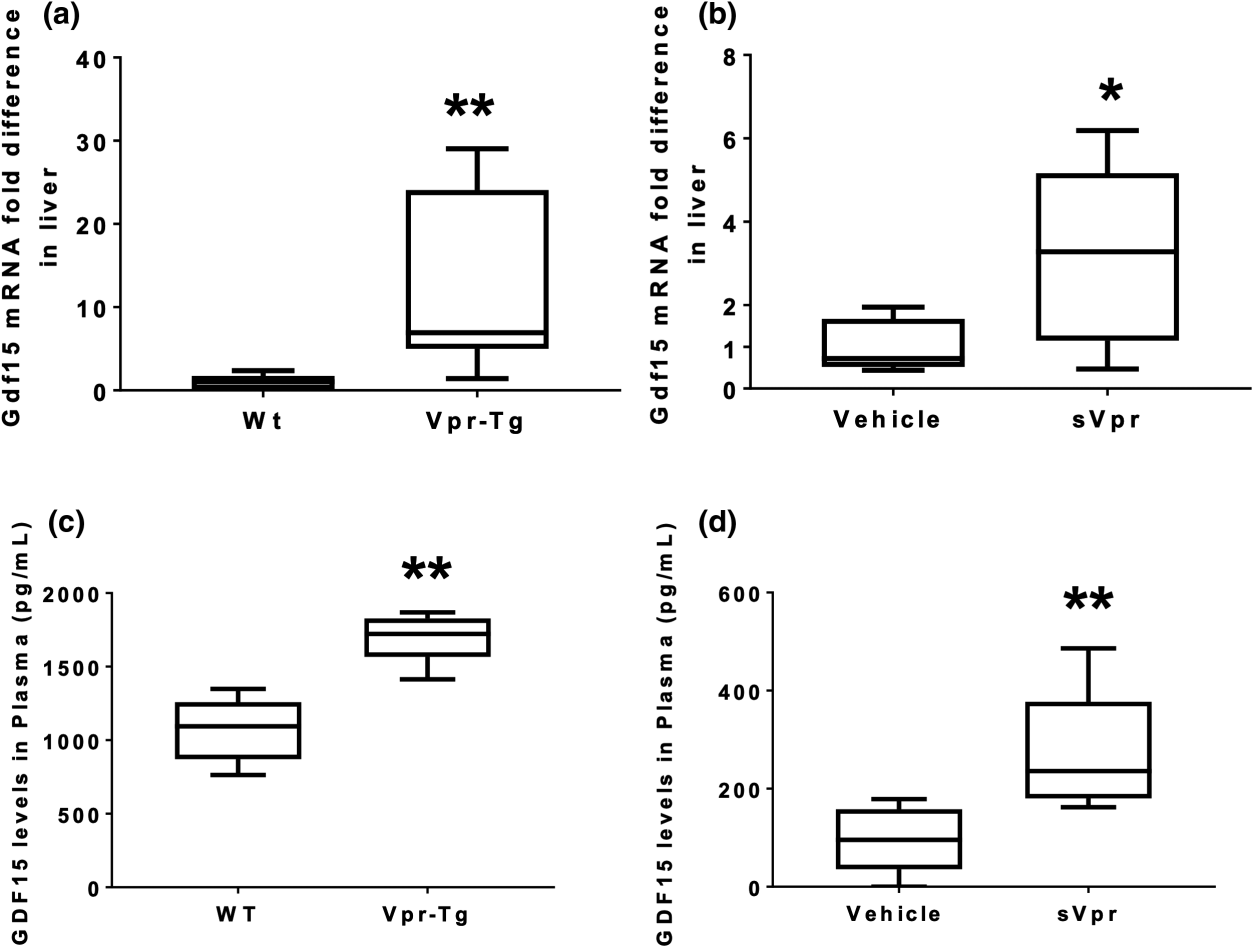
Increased mRNA levels of GDF15 in the liver and plasma of Vpr‐Tg and sVpr‐treated mice. (a) *Gdf15* mRNA expression is increased in the liver of Vpr‐Tg compared to WT mice (*p* = 0.002; *N* = 8 per group). (b) *Gdf15* mRNA expression is increased in the liver of sVpr‐treated compared to vehicle‐treated mice (*p* = 0.02; *N* = 8 per group). (c) Increased GDF15 plasma levels in Vpr‐Tg compared to WT mice (*p* = 0.002; *N* = 6 per group). (d) Increased GDF15 plasma levels in sVpr‐treated compared to vehicle‐treated mice (*p* = 0.009; *N* = 5–6 per group). Values are median with interquartile range. *p < 0.05, **p < 0.01, ***p < 0.001

Our retrospective analysis of RNA‐Seq data (Agarwal et al., 2013; Agarwal et al., 2017) uncovered strong induction of *Gdf15* mRNA in the liver of Vpr‐Tg mice. To further explore whether GDF15 is associated with metabolic disease in the Vpr mouse models, we measured *Gdf15* gene expression in the liver of male mice. Vpr‐Tg mice showed 13‐fold higher *Gdf15* mRNA expression in the liver than WT littermates (*p* = 0.002, Figure 1a) and 3‐fold higher *Gdf15* mRNA expression in the liver of sVpr‐treated mice compared to vehicle‐treated mice (*p* = 0.02, Figure 1b). To determine the implications of increased *Gdf15* gene expression in the liver, we measured plasma GDF15 levels in the Vpr mice. As predicted from the fatty liver phenotype (Agarwal et al., 2017) and hepatic *Gdf15* expression changes, Vpr mice displayed elevated plasma levels of GDF15: 1.6‐fold higher in Vpr‐Tg mice compared to WT littermates (*p* = 0.002; Figure 1c) and approximately 3‐fold higher in sVpr‐treated mice compared to vehicle‐treated mice (*p* = 0.009; Figure 1d).

### 3.2 PLWH harbor elevated levels of circulating Gdf15

**FIGURE 2:**
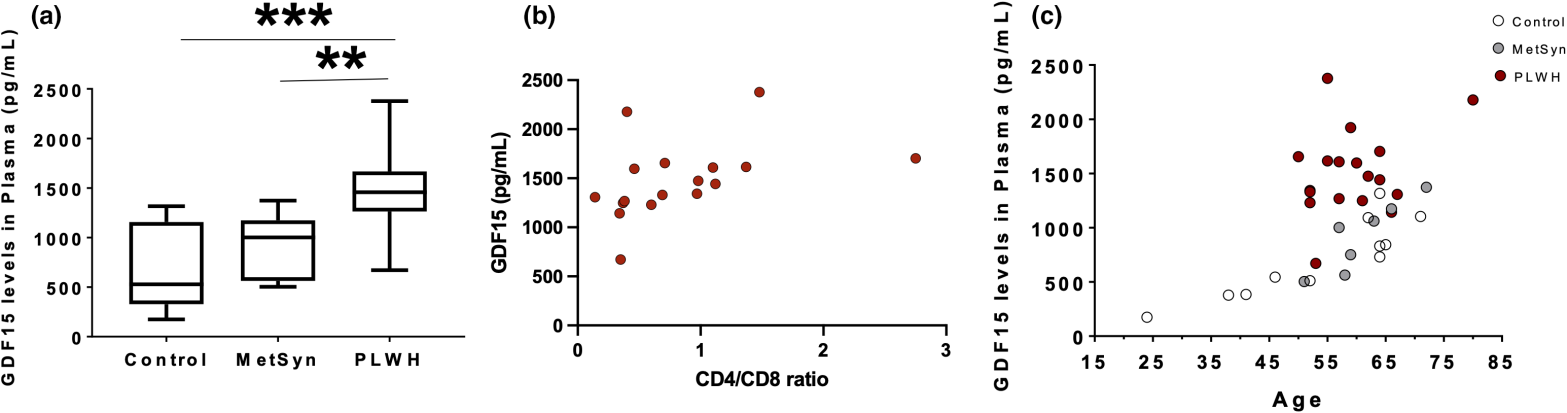
Correlates of GDF15 plasma levels in PLWH. (a) Increased GDF15 plasma levels in PLWH (*N* = 18) compared to HIV‐negative healthy controls (*p* = 0.0007; *N* = 6) and HIV‐negative MetSyn patients (*p* = 0.0014; *N* = 7). (b) GDF15 levels correlated positively with CD4/CD8 ratio in PLWH (Spearman correlation coefficient *r*_s_ =0.71, *p* = 0.002). (c) GDF15 levels correlated positively with age in the healthy controls (*R* ^2^ = 0.77, *p* = 0.0004) and persons with MetSyn (*R* ^2^ = 0.80, *p* = 0.007) but not in PLWH (*R* ^2^ = 0.09, *p* = 0.2351)

To establish whether plasma levels of GDF15 in PLWH differ from those of HIV‐negative persons, we compared plasma GDF15 levels of the PLWH with those in plasma samples from both healthy control men and obese men with MetSyn (Table 1). Similar to observations in Vpr mice, plasma levels of GDF15 were 2‐fold higher in the PLWH compared to controls (*p* = 0.0007) and 1.5‐fold higher compared to MetSyn subjects (*p* = 0.001; Figure 2a). Median value of GDF15 in control was 174.1 pg/ml (IQR: 327.7–1156); in MetSyn subjects was 1002 pg/ml (IQR: 563–1174) and in PLWH was 1458 pg/ml (IQR: 1263–1667) (Table 2).

**TABLE 2:** GDF15 levels in the study populations

	PLWH	HIV‐negative healthy controls	HIV‐negative metabolic syndrome patients
Number of subjects	18	6	7
GDF15 level (pg/ml)	1507 ± 391.6 (1458, 1263–1667)	671.4 ± 441.9 (528, 327.9–1156)	918.4 ± 322.7 y

Note

Values represent mean ± SD. Numbers in parenthesis represent mean and interquartile range (median, lower quartile–upper quartile). *p* values are based on Mann Whitney *U* test. Units of measurement of GDF15 levels are pg/ml.

### 3.3 Plasma GDF15 levels correlate with T cell subsets in PLWH

Chronic ART restores CD4^+^ T cell (CD4) levels in PLWH. Especially in those with lipodystrophy or MetSyn, these changes are accompanied by increased levels of stress‐associated hormones such as FGF21 (Domingo et al., 2010). We asked whether plasma GDF15 levels were also altered in relation to CD4 and CD8 T cell subsets. The PLWH showed a positive correlation of plasma GDF15 levels with CD4 count (Spearman's rho, *r*_s_ =0.62, *p* = 0.009; Table 3), an inverse correlation of plasma GDF15 levels with CD8 count (Spearman's rho, *r*_s_ = −0.56, *p* = 0.02; Table 3) and a strong positive correlation with the CD4:CD8 ratio (Spearman's rho, *r*_s_ =0.71, *p* = 0.002; Table 3; Figure 2b).

**TABLE 3:** Spearman correlation of GDF15 plasma levels with clinical parameters in PLWH

	*r*_s_‐value	*p*‐value
Age (years)	0.08597	0.73
Ethnicity	−0.02464	0.92
Body weight (kg)	−0.07327	0.77
Body mass index (BMI, kg/m^2^)	−0.03199	0.90
Glucose (mg/dl)	0.3348	0.19
Glycated hemoglobin (HbA1c, %)	−0.4454	0.23
Total cholesterol (mg/dL)	−0.2134	0.41
High density lipoprotein (HDL‐C, mg/dl)	−0.08621	0.41
Low density lipoprotein (LDL, mg/dl)	−0.1887	0.47
Triglycerides (TG, mg/dl)	−0.2465	0.34
Aspartate aminotransferase (AST, mg/dl)	−0.04712	0.86
Alanine aminotransferase (ALT, mg/dl)	−0.00491	0.99
White blood cell count (WBC, ×10^9^/L)	0.4794	0.06
CD4 count (cells/mm^3^)	0.6242	0.009
CD8 count (cells/mm^3^)	−0.5632	0.020
CD4:CD8 ratio	0.7083	0.002

### 3.4 Plasma GDF15 levels correlate with age in HIV‐negative subjects but not in PLWH

We performed additional correlation analyses of GDF15 with available metabolic measurements and demographics. Both HIV‐negative groups showed strong correlations of GDF15 levels with age (linear regression analysis *r* ^2^ = 0.77 and 0.80, respectively; *p* = 0.0004 and 0.007 respectively; Figure 2c) but this relationship was not apparent in the PLWH (linear regression analysis *r* ^2^ = 0; *p* = 0.24; Figure 2c).

## 4 DISCUSSION

Plasma levels of GDF15, a member of the transforming growth factor‐β superfamily, are increased in diabetes, cardiovascular disease, chronic inflammatory diseases, and NAFLD (Adela & Banerjee, 2015; Hsiao et al., 2000; Li et al., 2018; Moon et al., 2020). The purpose of this study was to understand how plasma GDF15 levels change in PLWH relative to HIV‐negative persons with and without MetSyn. Higher levels of GDF15 in a spectrum of inflammatory and metabolic diseases formed the prediction that PLWH harbor elevated levels of GDF15 in plasma.

We noted differences in the degree of elevation of GDF15 levels between the two Vpr mouse models that likely occur due to differences in the nature of Vpr expression (Agarwal et al., 2013). In the Vpr‐Tg model, transgenic expression of Vpr in *Pepck*‐positive tissues (adipose tissue, liver, and kidney) occurs through most of the life of the animal. In contrast, in the sVpr model, the synthetic protein is infused in young adult mice for two weeks. Differences in tissue‐specific gene and protein levels could occur due to the chronic versus short duration of the Vpr exposure strategies. Nonetheless, the energy balance phenotypes of both Vpr mouse models are very similar and recapitulate all critical aspects of HIV‐associated fatty liver disease in PLWH.

GDNF family receptor alpha‐like (GFRAL), a transmembrane receptor localized to the hindbrain (Emmerson et al., 2017; Mullican et al., 2017), binds GDF15 and mediates its satiety effects in rodents. Additional studies demonstrated that GDF15 critically mediates the appetite and weight loss effects of metformin (Day et al., 2019). GDF15 also reduces physical activity in rodents (Macia et al., 2012; Wang et al., 2013). Our finding of elevated levels of GDF15 protein in the plasma and mRNA expression in the liver of Vpr mice adds to a complex picture of the endocrine effects of GDF15. We recently showed that Vpr disrupts energy balance and thermogenic regulation of subcutaneous adipose tissue in mice (Agarwal et al., 2021). Vpr‐Tg mice cannot defend body temperature when exposed to cold and display lower energy expenditure at room temperature (Agarwal et al., 2021). Mullican et al. reported that the GDF15 effect on body mass is due to reduced food intake (increased satiety) rather than increased energy expenditure (Johnen et al., 2007; Mullican et al., 2017; Yang et al., 2017). While we have not rigorously investigated satiety in our mouse models, we have not observed significant differences in food intake or activity between the Vpr mice and their respective controls (Agarwal et al., 2021). Hence alternative mechanisms probably underlie energy balance changes occurring in parallel with elevated GDF15 in these mice. In our study, we used male mice, which show greater sensitivity to genetic and dietary interventions that cause fatty liver disease (Clegg & Mauvais‐Jarvis, 2018) and most faithfully model the human samples available to us. Additional studies are underway to test whether GDF15 levels in Vpr mice and PLWH are associated with fatty liver and resistance to the central nervous effects of GDF15 in male and female mice, similar to other hormone resistance paradigms in PLWH (Bourgi et al., 2018).

There is emerging evidence that GDF15 accumulates in the plasma of older adults (Alcazar et al., 2021; Kim et al., 2022; Yamaguchi et al., 2021). Linear regression analysis showed that GDF15 levels in the plasma of healthy controls and MetSyn HIV‐negative subjects correlate positively with age. There was no such association in the PLWH. Although the age nominally varied amongst the subjects by ten years, our data suggest that circulating GDF15 levels in PLWH are not dependent on age. PLWH develops age‐related comorbidities more frequently and at earlier ages than individuals without HIV (Rodriguez‐Penney et al., 2013; Schouten et al., 2014). Thus, higher GDF15 levels in PLWH occurring independently of age may mirror the confluence of older PLWH with the prevalence of advanced age‐related metabolic comorbidities, such as NAFLD, and significantly raises the disease burden in PLWH.

GDF15 evolved as a signal of cellular stress and represents a biomarker of disorders of mitochondrial function. The lack of a relationship of GDF15 levels with age in the PLWH could partially reflect the phenomenon of accelerated aging in PLWH on ART, with premature development of age‐related diseases. It has been demonstrated that ART accelerates mitochondrial aging through the clonal expansion of mtDNA mutations and thereby induces mitochondrial dysfunction (Payne et al., 2011). Furthermore, ART induces hallmarks of aging, including persistent low‐grade inflammation and senescence in various cell types, even after relatively short exposures (Caron et al., 2008). We additionally saw a positive correlation between GDF15 plasma concentration and circulating CD4 count and CD4:CD8 ratio, which could reflect the need for immune reconstitution on ART to mount sufficient stress responses. Furthermore, insight will be gained from the measurement of GDF15 levels in PLWH after contemporary ART and in the context of accelerated age‐related disorders such as sarcopenia and NAFLD.

GDF15 secretion in rodents derives from energetic stress on peripheral tissues, including the liver (Lockhart et al., 2020). Outside of GFRAL requirements for GDF15 effects on energy balance, the downstream signaling mechanisms of GDF15 and the regulation of its tissue expression have not been well defined. Furthermore, whether GDF15 serves a causal role in metabolic diseases or simply reflects energetic stress remains controversial. Key intracellular proteins governing GDF15 expression include transcription factors of the integrated stress pathway such as ATF4 (Patel et al., 2019), whose regulation becomes altered in human and rodent NAFLD (Puri et al., 2008; Seo et al., 2009). Our studies of Vpr mice allow testing of possible mechanisms supporting GDF15 secretion and action. Plausible mechanisms based on known stimuli of GDF15 expression (Hsiao et al., 2000) in the liver include Vpr‐induced mitochondrial dysfunction (Agarwal et al., 2021) and overall hepatocellular stress induced by multiple mechanisms leading to NAFLD (Agarwal et al., 2017) in the Vpr mice. It is also possible that an unidentified transcriptional pathway linked to Vpr's promiscuous ability to co‐regulate host genes related to lipid/sterol metabolism or oxidative stress (Agarwal et al., 2017, 2021) or to inhibit PPARγ (Agarwal et al., 2013) could be responsible. In addition to MetSyn, plasma GDF15 may also be a biomarker of pulmonary hypertension and heart failure risk in PLWH with low‐level viremia (Elvstam et al., 2019; Scherzer et al., 2018; Secemsky et al., 2015). Whether GDF15 mediates maladaptive or adaptive responses to organ stress in the liver or elsewhere remains an open question.

Another limitation of our human study is the lack of biological and ethnic diversity. Accumulating evidence demonstrates that the prevalence and severity of NAFLD are affected by sex and age (Vilar‐Gomez et al., 2018). In HIV‐negative adults, NAFLD prevalence is higher in men than in pre‐menopausal women (or aged ≤ 50–60 years), while NAFLD tends to become more common in women after menopause or after age 50 (Long et al., 2018). All plasma samples in the study were male, which does not allow testing of female influences on GDF15 levels in PLWH. Estrogen inhibits HIV transcription (Das et al., 2018) and may engender the differences in MetSyn prevalence between men and women living with HIV. Nonetheless, there remains little information regarding the mechanisms that contribute to the higher prevalence and severity of NAFLD among PLWH and the role of gender, race, and ethnicity on these risk factors (Lim & Bernstein, 2018; Soti et al., 2018). Unfortunately, most of the available data on NAFLD in PLWH is disproportionately from studies of male subjects (Maurice et al., 2017). Considering that women and minorities living with HIV have a greater prevalence of obesity and are at greater risk to experience poorer outcomes and increased weight gain from HIV treatment (Erlandson et al., 2016; Soti et al., 2018), there is a clear need for biomarker studies of NAFLD in these communities of PLWH.

Ethnic differences in GDF15 have been reported in acute coronary syndrome, with median GDF15 concentrations in the range of 1244–1835 ng/dl in Europeans (Zhang et al., 2016) and 650 ng/dl in Asians (Lin et al., 2014). Conversely, in a multiethnic cohort of middle‐aged Dallas County adult residents, 53% of African American subjects had GDF15 levels equal to or greater than 1800 ng/dl, whereas only 17% of White subjects had GDF15 levels in that range (Rohatgi et al., 2012). Although our study population was predominantly White and had the same number of African American subjects in the PLWH and MetSyn groups, larger studies in PLWH and controls would be needed to assess the role of ethnicity in GDF15 expression in PLWH. In addition, the specific role of ART on GDF15 secretion was not evaluated in this cohort of PLWH because the current standard of care is to treat all PLWH with ART.

GDF15 levels are elevated in NAFLD in proportion to disease severity (Koo et al., 2018). Specific HIV‐related risk factors (e.g., effects of persistent viral factors such as Vpr, immune activation, and antiretroviral drugs) may result in an altered course of liver disease and sustained GDF15 secretion. Development and progression of steatosis and the risk for progression towards NASH are more pronounced in patients receiving ART (Bischoff et al., 2021). To our knowledge, this is the first study that measured circulating GDF15 in PLWH and orthogonal mouse models. Our data suggest that GDF15 levels are elevated in PLWH on suppressive ART and that Vpr could be a specific, causative HIV‐associated factor that either directly modulates its expression in the liver or provokes its expression and secretion as a result of persistent hepatocellular stress from HIV‐related risk factors.

## CONFLICT OF INTEREST

The authors declare no conflicts of interest.

## AUTHOR CONTRIBUTIONS

A.B., J.E.L., and S.M.H. conceptualized the study. N.A, A.B., C.E.R‐B., and S.M.H. designed experiments. J.E.L, H.W., and R.V. provided clinical specimens. N.A. performed ELISA and qPCR studies. N.A, A.B., C.E.R‐B., and S.M.H wrote the manuscript with editorial input from all authors. All authors provided interpretations of the data. All authors have approved the submitted manuscript.

## ETHICS STATEMENT

This study was approved by the IRB at UCLA and UTHSC‐Houston and was carried out in accordance with the Declaration of Helsinki. Written informed consent was obtained from all participants prior to their participation in the study.
